# Does changing clinicians’ attitudes toward self-harm reduce coercive interventions in mental health inpatient settings? A quasi-experimental study of staff training

**DOI:** 10.1186/s40479-026-00346-2

**Published:** 2026-08-10

**Authors:** Kristine Høst Poulsen, Carsten Hjorthøj, Bo Bach, Erik Simonsen, Mickey T. Kongerslev

**Affiliations:** 1 Mental Health Services East, Central and West Zealand Hospital, Smedegade 16, Roskilde, 4000 Region Zealand, Roskilde Denmark; 2 Department of Psychology, University of Southern Denmark, Odense, Denmark; 3 Copenhagen Research Center for Mental Health - CORE, Bispebjerg and Frederiksberg Hospital, Copenhagen, Denmark; 4 Section of Epidemiology, Department of Public Health, University of Copenhagen, Copenhagen, Denmark; 5 Mental Health Services West, Central and West Zealand Hospital, Slagelse, Region Zealand Denmark; 6 Department of Psychology, University of Copenhagen, Copenhagen, Denmark; 7 Department of Clinical Medicine, University of Copenhagen, Copenhagen, Denmark

**Keywords:** Self-harm, Non-suicidal self-injury, Staff training, Coercion, Restraint, Inpatient psychiatry, Implementation, Attitudes, Interrupted time series

## Abstract

**Background:**

Staff education may improve clinicians’ attitudes toward self-harm, but whether this translates into reduced coercion is unclear. We evaluated a regional strategy to improve care for self-harming patients and its impact on staff attitudes and coercive interventions across services.

**Methods:**

Quasi-experimental dual design: (a) interrupted time-series of registry data on coercive interventions (Jan 2021–Dec 2024) and (b) three-wave pre–post surveys using the revised 17-item Self-Harm Antipathy Scale (SHAS-DR) at baseline, 2 months, and 8 months post-training. Change points: June 2022 (strategy announcement) and June 2023 (training implementation).

**Results:**

In registry data, coercive interventions declined after the strategy announcement (level IRR 0.39, 95% CI 0.21–0.73) with no subsequent trend change. A temporary increase occurred at the training point (level IRR 2.58, 95% CI 1.57–4.25), consistent with implementation disruption. Reductions were largest for mechanical restraints, particularly among self-harming patients, while rapid tranquillization remained most frequent. Annual coercive episodes fell from 1,280 (2021) to 409 (2024). Staff attitudes improved and were sustained. Mean SHAS-DR decreased from 40.0 (SD 10.8) at baseline to 34.8 (SD 7.4) at 2 months and 35.4 (SD 8.2) at 8 months; baseline-to-follow-up changes were significant (*p* ≤ 0.001).

**Conclusions:**

The strategy coincided with an immediate reduction in coercion and a transient rise during training, while staff attitudes reflected reduced antipathy toward people who self-harm. Sensitivity analyses suggested changes were not driven by high-frequency patients. Sustained reductions likely require continued organizational and relational supports.

**Trial registration:**

Not applicable.

**Supplementary information:**

The online version contains supplementary material available at 10.1186/s40479-026-00346-2.

## Background

In inpatient mental health care, self-harm (including non-suicidal self-injury) is common and may lead to coercive interventions such as mechanical restraint and rapid tranquillization. Yet, it remains unclear whether staff training and improved attitudes translate into measurable reductions in coercion.

Self-harm is a common and clinically challenging presentation in inpatient mental health care [1, 2]. Staff are often required to balance empathic engagement with immediate safety management, and episodes of self-harm may evoke fear, frustration, and moral judgement that shape how the behavior is interpreted and managed [3–5]. 

For decades, educational initiatives have sought to improve staff knowledge, confidence, and attitudes toward self-harm [6–8]. Training programs, often Dialectical Behavioral Therapy (DBT)-informed and/or co-produced with service users, tend to yield modest but consistent improvements in attitudes and perceived competence [9–11]. However, it remains uncertain whether attitudinal change translates into sustained changes in ward-level practice, including reduced self-harm and reduced use of coercive interventions. Reviews indicate that effects of ward-based interventions are frequently short-lived and that institutional culture and organizational conditions may shape coercive practice more strongly than training alone [12, 13].

Coercive measures such as mechanical restraints or rapid tranquillization may ensure immediate safety but risk reinforcing a cycle of dysregulation on both sides of the therapeutic relationship [2, 3]. Despite guidelines emphasizing relational safety and therapeutic risk-taking [14, 15], staff often report moral tension and uncertainty [4, 11]. Coercion can feel punitive, traumatic, or shaming to patients, eroding trust and safety [3, 16].

Staff attitudes are therefore clinically relevant: they shape how responsibility is attributed and which responses are considered feasible in practice [17, 18].

In 2022, Mental Health Services in Region Zealand, Denmark, introduced a comprehensive Regional Self-Harm Strategy aimed at strengthening consistent, relational care for individuals who self-harm across services. The strategy emphasizes validation, collaborative crisis planning, and structured de-escalation, and was implemented system-wide; structured staff training was rolled out in 2023. The strategy draws on established approaches to relational safety and emotion regulation (e.g., Safewards and DBT-informed principles [9, 19, 20]. As a system-wide, multi-component quality-improvement initiative, its impact is expected to depend not only on training content but also on contextual and implementation processes [21]. Evaluating both staff attitudes and system-level coercion outcomes may therefore clarify whether improvements in attitudes coincide with measurable changes in coercive practice.

### Aim

We examined whether implementation of the regional self-harm strategy and subsequent staff training were associated with changes in:

1. **Staff attitudes:** Reduced antipathy toward people who self-harm.
2. **Coercion:** Reductions in the use of coercive interventions in adult inpatient units, overall and among episodes involving patients with versus without self-harm.

## Methods

### Reporting statement

Reporting of the observational components adheres to the STROBE guidelines [22]. Reporting of the interrupted time-series analysis follows established methodological guidance for ITS studies [23]. Reporting of the implementation components follows the StaRI statement [24].

### Study design

We used a quasi-experimental dual design with two complementary components:

- an interrupted time-series (ITS) analysis of monthly registry data on coercive interventions from January 2021 to December 2024, and
- a three-wave pre–post assessment of staff attitudes toward self-harm.

Two predefined milestones structured the analysis: June 2022 (strategy announcement) and June 2023 (post-training). The intervention was implemented system-wide, which made randomization infeasible. The design followed guidance for quasi-experimental evaluation in implementation research [25]. Because the regional self-harm strategy was implemented system-wide and comprised interacting components beyond training, it can be conceptualized as a complex intervention in which outcomes depend on the interplay between content, context, and implementation processes [21, 26, 27]. The overall procedure and timeline are illustrated in Supplementary Fig. 1.

### Setting and participants

The study included all five adult inpatient units within Mental Health Services East, Region Zealand, Denmark. Each of the five units had a capacity of approximately 13 beds, although the exact number varied across units. One unit served as a “double unit” at the time of the study and therefore had a capacity of 2 × 13 beds. In addition, the psychiatric emergency unit had 12 beds, including 8 open beds and 4 closed beds in the high-observation area. Overall, the combined bed capacity across units was 81. All five units are authorized under Danish law to apply coercive measures in accordance with the Act on the Use of Coercion in Psychiatry [28]. No major organizational changes were introduced during the study period that would be expected to independently affect coercion rates.

Eligible participants were clinical staff with direct patient contact, including nurses, social- and healthcare assistants, social educators, psychologists, occupational and physical therapists, and psychiatrists. Non-clinical personnel were excluded. A total of 187 staff participated in the training, and 182 completed the baseline attitude questionnaire. Of the 182 baseline respondents, 113 provided valid attitude data at two or more time points. The ITS dataset comprised fully de-identified monthly counts of coercive interventions from the same units.

### Intervention and training program

The intervention translated the Regional Self-Harm Strategy (Region Zealand, 2022) into a structured training and education program for inpatient staff. The Strategy is a cross-sector framework that outlines shared principles for the assessment, management, and follow-up of non-suicidal self-harm. It draws on evidence and best practice, including the Safewards model of relational safety [19, 20], the recovery-oriented Tidal Model [29, 30], and DBT-informed principles of emotion regulation [9]. The strategy emphasizes dialogue, validation, collaborative crisis planning, and the Ten Interventions for Self-Harm, which provide practical guidance for relational safety and structured de-escalation (see Supplementary Table 8).

A five-hour training program was delivered to all inpatient staff between May and June 2023 (see Supplementary Table 7). The first author designed and delivered the curriculum, drawing on input from a person with lived experience of severe self-harm during the design phase. The curriculum introduced key elements of the Regional Strategy and focused on strengthening clinical competence and consistency. Implementation fidelity was supported through standardized training materials, local trainers, and structured supervision, and all five inpatient units completed the training as planned.

Content covered in the training:

- theoretical and clinical understandings of self-harm and non-suicidal self-injury [1, 31];
- principles of emotional regulation and relational communication, with Strain Psychology (Belastningspsykologi) used to discuss emotional strain in Danish mental health practice [32];
- therapeutic risk-taking as one component within the broader strategy [14]; 
- legal and ethical aspects of coercion in Danish inpatient care [28] and
- Dialectical Behavior Therapy (DBT)-informed clinical tools for emotion regulation and crisis response [9].
- Each unit appointed two local trainers who received additional instruction and supported implementation through supervision, consultation, and case-based reflection.

### Definitions and terminology

In this paper, self-harm refers to deliberate self-inflicted injury or behavior, with or without suicidal intent, consistent with clinical terminology in Danish mental health services. The regional self-harm strategy was primarily developed to address non-suicidal self-injury (NSSI), defined as repetitive self-inflicted harm without suicidal intent [1, 31]. However, in routine clinical documentation and staff practice, distinctions between suicidal and non-suicidal self-harm are often blurred. The present study therefore uses self-harm as an inclusive term, while recognizing that the intervention primarily targeted NSSI-related behavior.

## Measures

### Staff attitudes

Staff attitudes toward self-harm were assessed using the Self-Harm Antipathy Scale (SHAS), a 30-item questionnaire developed to measure clinicians’ attitudes toward deliberate self-harm [6]. At all three time points, participants completed the 30-item Danish translation of the SHAS (SHAS-D), rated on a 7-point scale. Positively worded items were reverse scored so that lower scores indicate more positive attitudes.

A Danish psychometric evaluation of the SHAS-D demonstrated that the original 30-item structure did not show adequate structural validity, whereas a revised 17-item scoring (SHAS-DR) provided good model fit, acceptable reliability, and conceptually coherent subscales for use in Danish mental health care [33]). Accordingly, all attitudinal analyses in the present study used SHAS-DR scoring derived from the SHAS-D responses. SHAS-DR total scores were calculated as the sum of the 17 retained items. When two or fewer of the 17 items were missing, prorated scores were computed; otherwise, the questionnaire was excluded from analysis.

### Coercive interventions

Monthly counts of coercive interventions (mechanical restraint with belts, straps, or holding, and rapid tranquillization) were extracted from the national coercion registry (SEI) by a senior Training and Development Instructor responsible for coercion reduction in Mental Health Services East, who was not part of the research group. All 2,641 coercive episodes recorded in the registry across the four-year study period (2021–2024) received a valid SHP/NSHP classification. Classification followed a two-step procedure: a senior training and development instructor with direct knowledge of the patient population first reviewed all episodes and cross-referenced SEI data with coercion protocols and consulted unit staff to confirm whether the patient was known to engage in self-harm; the first author then independently verified each classification by reviewing clinical documentation in the electronic health record (Sundhedsplatformen) under authorized access. This dual-verification procedure supports the completeness and validity of the classification dataset.

Data remained pseudonymized throughout.

The analytic dataset contained no directly identifiable patient information. The primary ITS analyses were based on monthly aggregated counts (overall and stratified by coercion type and SHP/NSHP status). Where patient-level information was required for sensitivity and concentration analyses, data were handled in pseudonymized form and are reported only as aggregated summaries.

## Procedures

### Administration of the SHAS-D

The SHAS-D was administered at three time points: baseline (immediately before training), two months, and eight months. At baseline, participants provided demographic information and written informed consent. The first author assigned each participant a unique number for pseudonymized follow-up. At two and eight months, questionnaires were distributed by ward managers and local trainers. Completed forms were returned to the first author and entered into the dataset. SHAS-DR scoring was applied to the SHAS-D responses as described above.

### Sensitivity and concentration procedures

To assess whether observed reductions in coercion were driven by a small number of high-frequency patients, we conducted concentration sensitivity analyses using pseudonymized patient-level registry data from 2021 to 2024. For each year (and separately for each coercion type), patients were ranked by number of coercive episodes, and we calculated the proportion of episodes accounted for by the top 10% and top 5% most frequently exposed patients.

We then constructed three alternative scenarios: heavy10, in which episodes contributed by the top 10% of patients were excluded; heavy05, in which episodes contributed by the top 5% were excluded; and cap-1, in which each patient contributed a maximum of one coercive episode per year (i.e., counting unique patient-years).

### Statistical analysis

Segmented regression was used to estimate level and slope changes at the two milestones. Parameters included time, post₁, trend₁, post₂, and trend₂. Post₁ and post₂ represent immediate level changes after each milestone; trend₁ and trend₂ represent changes in monthly slope. Primary models used Poisson regression with robust standard errors. Negative-binomial models were fitted when overdispersion was detected (χ^2^/df > 1.5). Calendar-month fixed effects adjusted for seasonality. Results are reported as incidence-rate ratios (IRRs, 95% CI). Analyses were repeated separately for SHP and NSHP episodes. Concentration metrics (share of episodes contributed by the top 10% and top 5% of patients, and the heavy10/heavy05/cap-1 scenarios described above) were calculated to assess robustness.

Changes in staff attitudes were analyzed using linear mixed-effects models with random intercepts, using the validated SHAS-DR total score as the outcome measure, with paired t-tests as complementary checks. Residual plots were inspected for autocorrelation. All analyses were conducted in Stata 18 (StataCorp LLC, College Station, TX, USA).

## Results

### Response rates and attrition

A total of 187 staff attended the training sessions, and 182 completed the baseline attitude questionnaire. 165 met the SHAS-DR completeness rule (≤2 missing of the 17 scored items) and were included in cross-sectional descriptive baseline summaries. Ninety participants responded at two months and seventy-four at eight months. The longitudinal analytic sample included 113 participants who provided valid SHAS-DR data for at least two time points; fifty-one staff completed all three waves. Attrition was highest at eight months and reflected shift schedules, staff turnover, and routine survey fatigue in high-intensity inpatient settings.

### Participant characteristics

Participant characteristics appear in Table 1. At baseline, seventy-eight percent identified as female, twenty-one percent as male, and one percent did not report gender. The largest professional groups were social- and healthcare assistants (48%) and nurses (25%). Smaller groups included social educators, physicians, occupational therapists, social workers, psychologists, and support staff.

**Table 1:** Participant professions and gender distribution at baseline and analytic sample

Profession	Baseline n (%)	Female n (%)	Male n (%)	Included in analysis n (%)	Female n (%)	Male n (%)
Social- and healthcare assistant	87 (47.8)	69 (79.3)	17 (19.5)	55 (48.7)	45 (81.8)	9 (16.4)
Nurse	45 (24.7)	40 (88.9)	5 (11.1)	31 (27.4)	27 (87.1)	4 (12.9)
Social educator	15 (8.2)	12 (80.0)	3 (20.0)	10 (8.8)	8 (80.0)	2 (20.0)
Doctor	9 (4.9)	5 (55.6)	4 (44.4)	2 (1.8)	1 (50.0)	1 (50.0)
Mental health care attendant	6 (3.3)	3 (50.0)	3 (50.0)	2 (1.8)	1 (50.0)	1 (50.0)
Occupational therapist	5 (2.7)	3 (60.0)	2 (40.0)	3 (2.7)	2 (66.7)	1 (33.3)
Social worker	5 (2.7)	4 (80.0)	1 (20.0)	5 (4.4)	4 (80.0)	1 (20.0)
Psychologist	3 (1.6)	2 (66.7)	1 (33.3)	1 (0.9)	0 (0.0)	1 (100.0)
Support staff	4 (2.2)	4 (100.0)	0 (0.0)	4 (3.5)	4 (100.0)	0 (0.0)
Physical therapist	2 (1.1)	0 (0.0)	2 (100.0)	–	–	–
**Total**	182 (100)	142 (78.0)	38 (20.9)	113 (100)	92 (81.4)	20 (17.7)

*Notes:* Baseline *n* = 182; analytic sample = 113 staff with ≥2 valid SHAS time points. Percentages may not sum exactly due to rounding

## Staff attitudes toward self-harm

### Descriptive results

**Fig. 1:**
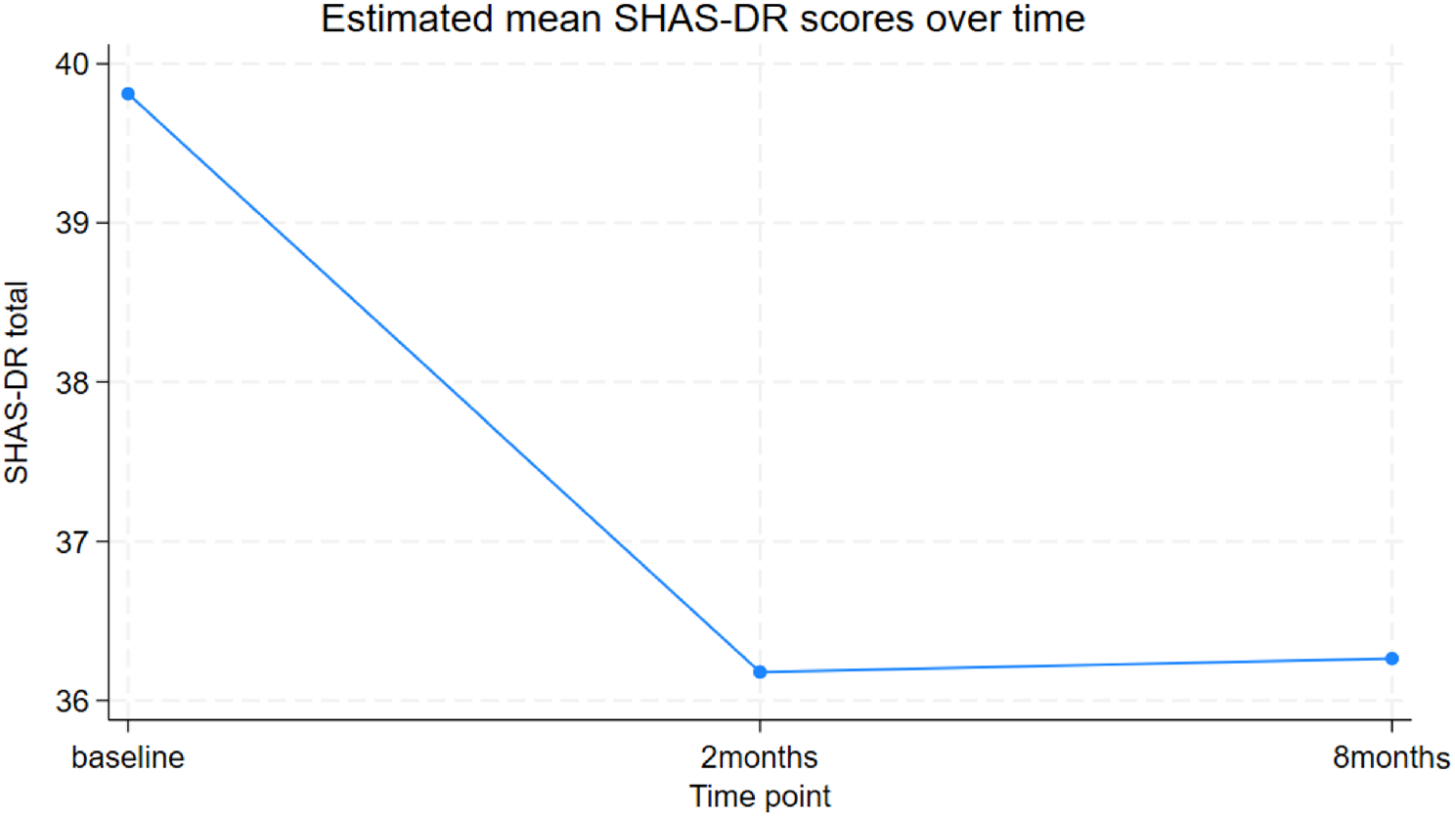
Estimated mean SHAS-DR scores with 95% confidence intervals. *Notes:* lower scores indicate more positive staff attitudes toward self-harm. Mixed-effects estimates (*n* = 113) showed a decrease from 39.81 at baseline to 36.18 at two months, with scores remaining stable at 36.26 at eight months

Descriptive results appear in Table 2, paired comparisons in Table 3, and mixed-effects estimates in Table 4. Estimated means with 95% confidence intervals are visualized in Fig. 1.

**Table 2:** Self-harm antipathy Scale – Danish revised (SHAS-DR) scores before and after training

Time point	n	Mean (SD)	Change from baseline
Baseline	165	40.0 (10.8)	–
2 months	88	34.8 (7.4)	−5.2
8 months	72	35.4 (8.2)	−4.6

*Notes:* Lower SHAS-DR scores = more positive attitudes toward people who self-harm. Change from baseline = raw mean difference

**Table 3:** Within-person change in SHAS-DR scores following training

Comparison	n pairs	Mean difference	95% CI	p
Baseline → 2 months	82	3.20	[1.78, 4.61]	<.001
Baseline → 8 months	64	2.94	[1.23, 4.65]	.001

*Notes:* Paired-sample t-tests. Positive values indicate higher antipathy at baseline relative to follow-up (i.e., improvement). CI = confidence interval

**Table 4:** Linear mixed-effects model of SHAS-DR scores (*n* = 113)

Effect	Estimate (b)	SE	95% CI	p
Baseline (intercept)	39.81	0.77	[38.31, 41.31]	<.001
2 months vs baseline	−3.63	0.67	[−4.95, −2.32]	<.001
8 months vs baseline	−3.55	0.74	[−4.99, −2.11]	<.001

*Notes:* Random-intercept model; negative b = improvement (lower SHAS-DR scores). SE = standard error; CI = confidence interval

Mean SHAS-DR total scores decreased from 40.0 (SD = 10.8) at baseline to 34.8 (SD = 7.4) at two months and 35.4 (SD = 8.2) at eight months, reflecting reduced antipathy toward people who self-harm, particularly a decline in morally judgmental and blame-oriented perceptions, as indicated by the largest raw-score improvement on the Judgmental Perception subscale. It should be noted that the cross-sectional means in Table 2 reflect both within-person change and changes in sample composition across waves. The within-person estimates in Tables 3 and Table 4 show more moderate improvements of approximately 3 points, suggesting that part of the reduction in mean scores reflects differential attrition of participants with initially higher antipathy scores rather than change within individuals.

A similar pattern was observed across the three SHAS-DR subscales, with the largest raw-score improvement seen in *Judgmental Perception*.

### Paired comparisons

Among participants with matched data, SHAS-DR total scores declined significantly from baseline to two months (mean difference = 3.20, 95% CI [1.78, 4.61], *p* < 0.001) and from baseline to eight months (mean difference = 2.94, 95% CI [1.23, 4.65], *p* = 0.001). There was no significant difference between two and eight months (mean difference = 0.41, *p* = 0.60), indicating that the initial improvement was maintained without further change. These findings correspond to moderate effect sizes at both follow-up points.

### Mixed-effects models

Linear mixed-effects models confirmed these changes. Compared with baseline, SHAS-DR scores were 3.63 points lower at two months (*b* = −3.63, SE = 0.67, *p* < 0.001) and 3.55 points lower at eight months (*b* = −3.55, SE = 0.74, *p* < 0.001). The model-implied means were 39.81 at baseline, 36.18 at two months, and 36.26 at eight months (Fig. 1). Substantial between-person variability was indicated by the random intercept (Var[ID] = 79.23).

Together, the results indicate that structured staff training produced moderate, statistically robust, and sustained improvements in staff attitudes toward people who self-harm.

### Coercive interventions

**Fig. 2:**
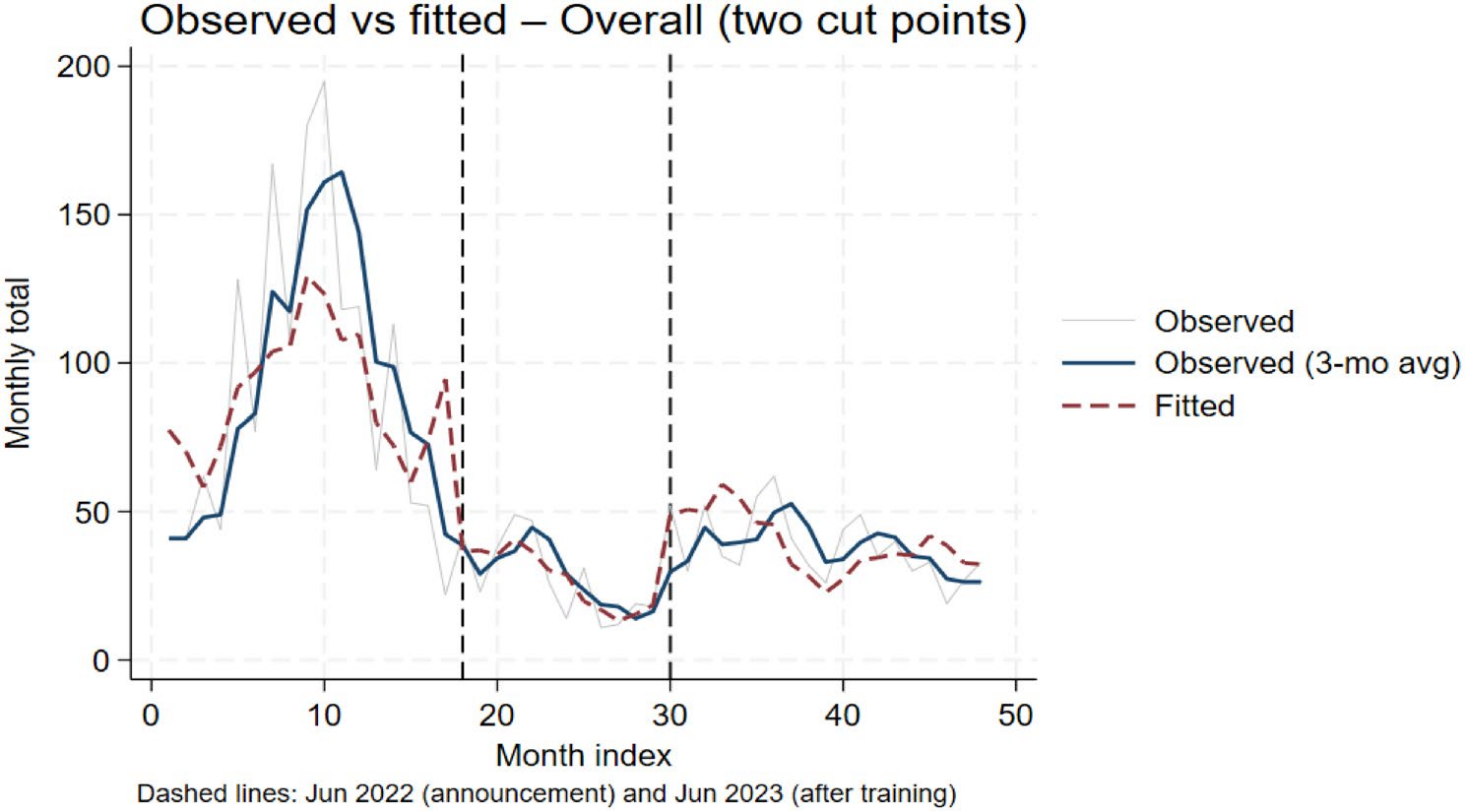
Monthly coercive interventions, January 2021–December 2024. *Notes:* the gray line represents observed monthly counts; the navy line shows the 3-month moving average. Vertical dashed lines mark June 2022 (strategy announcement) and June 2023 (post-training). The step-down after June 2022 illustrates the immediate policy impact, while the smaller rise in June 2023 reflects a transitional effect

Figure 2 shows trends in monthly coercive episodes, with segmented regression results in Table 5 and annual totals in Table 6. Total coercive episodes declined from 1,280 in 2021 to 543 in 2022 (a 58% reduction). Annual totals then stabilized and were identical in 2023 and 2024 (409 episodes in each year).

**Table 5:** Segmented regression of monthly coercive interventions (ITS model)

Parameter	IRR	95% CI	p
Time (months pre)	1.00	0.94–1.07	0.939
Level change at June 2022	0.39	0.21–0.73	0.003
Trend change after June 2022	0.94	0.86–1.03	0.182
Level change at June 2023	2.58	1.57–4.25	<0.001
Trend change after June 2023	1.03	0.97–1.09	0.328

*Notes:* Poisson regression with robust SEs (NB fallback). IRR < 1 = decrease; >1 = increase. Model includes calendar-month fixed effects. Milestones = June 2022 (strategy) and June 2023 (training)

**Table 6:** Annual totals of coercive interventions across all inpatient units

Year	Total episodes
2021	1 280
2022	543
2023	409
2024	409

*Notes:* Aggregated across five adult inpatient units, Mental Health Care Services East, Region Zealand. Totals reflect registry counts; 58% reduction from 2021 to 2022 followed by stabilization

Mean monthly episodes decreased from 93 before the strategy (January 2021 to May 2022) to 28 during the policy phase (June 2022 to May 2023) and then increased to 38 after training (June 2023 to December 2024).

### Interrupted time-series results

Segmented regression identified a large immediate reduction at the time of the strategy announcement (IRR = 0.39, 95% CI [0.21, 0.73], *p* = 0.003). The slope did not change after this point (IRR = 0.94, *p* = 0.182). At the training point, a temporary increase appeared (IRR = 2.58, 95% CI [1.57, 4.25], *p* < 0.001), consistent with short-term implementation-related disturbance rather than an effect of the training itself. The slope again remained unchanged (IRR = 1.03, *p* = 0.328). The overall pattern reflected two step changes rather than gradual trends.

#### By coercion type

Mechanical restraint showed the largest reductions. Between 2021 and 2024, use of belts decreased by 74%, straps by 81%, and holding by 82%. Rapid tranquillization decreased by 51% but remained the most frequent coercive measure. Full values appear in Supplementary Table 1.

#### By NSSI status (SHP vs. NSHP)

The composition of coercion shifted over time. In 2021, self-harming patients (SHP) accounted for most episodes (960 vs. 320 non-self-harming patients (NSHP)). By 2023 and 2024, NSHP episodes exceeded SHP episodes. Stratified ITS plots followed the same temporal pattern as the overall series. Supplementary Tables 1–3 provide full results, and Supplementary Table 6 shows coercion type by patient status.

### Sensitivity analyses

Heavy-patient analyses examined whether reductions in coercion were driven by a small group of frequently restrained individuals. Across all years, the ten percent most frequently restrained patients accounted for 52 to 58% of episodes, and the top five percent accounted for 35 to 41%. These proportions were stable. Removing the heavy10 group changed annual totals by less than five percent, and a cap-1 analysis, which limited each patient to one episode per year, showed a similar trajectory. Concentration was highest for mechanical restraint and lowest for rapid tranquillization. Full results appear in Supplementary Tables 4 and Table 5.

These findings indicate that the decline in coercion reflected a broad change in clinical practice rather than shifts among a small number of high-frequency patients.

## Discussion

Attitudes improved significantly and staff antipathy toward people who self-harm was reduced, with improvements remaining stable at eight months. Coercion fell sharply after the strategy announcement and stabilized at this lower level over the study period. A temporary rise occurred during the training period but did not alter long-term trends. Mechanical restraints showed the largest reductions. Rapid tranquillization remained the most frequent coercive measure. The number of coercion episodes involving self-harming patients declined over time. Together, the results suggest that improved attitudes are important but that sustained reductions in coercion also require ongoing structural and organizational support.

Because this was a system-wide quality improvement initiative without a control group (i.e. another mental health service), firm causal inferences are not possible. Even so, the temporal specificity of the changes, the pre-specified milestones, and the robustness of the sensitivity analyses support the interpretation that the strategy and associated training were linked to real behavioral change rather than to secular trends.

### Interpretation and theoretical implications

The immediate decline in coercion after the strategy announcement may represent an organizational “unfreezing” in Lewin’s sense [34], an early phase of cultural change initiated by leadership attention and a shared focus on self-harm rather than by new skills alone [14, 15, 25]. Similar patterns have been observed in other improvement efforts, where symbolic commitment and policy emphasis create initial movement, while training and tools later provide structure and language for changes that have already begun [23, 25].

Importantly, the regional self-harm strategy was not a single training package but a multi-component, cross-sector framework. It emphasized validation, collaborative crisis planning, and structured de-escalation, and it drew on established approaches to relational safety and emotion regulation [9, 19, 20, 29, 30]. In this light, the drop in coercion at the time of the strategy announcement may reflect changes in shared meaning and expectations—how self-harm is framed and what “safe care” is taken to entail—while the later training period may have represented a transitional phase in which new relational routines were rehearsed and consolidated under real-world constraints [14, 21, 23, 25].

From an affective and sociological perspective, the findings can be read through the lens of emotional labor and social defense theory. Hochschild [35] described how professionals regulate and display emotion according to institutional norms, transforming fear and frustration into calm and care, yet this deep acting rarely counts as technical expertise within systems that privilege observable procedures. Menzies [36] conceptualized institutions as social defenses against anxiety, where routines and hierarchies contain collective fear. When a self-harm strategy invites staff to replace coercive procedures with relational dialogue, these defenses are disturbed, and new emotional and moral capacities are required. The temporary rise in coercion during the training period may represent a defensive rebound, a short return to familiar control practices under strain rather than a failure of the strategy itself [23, 36]. Short-term increases around implementation points are well described as transition effects or implementation dips, where existing routines are unsettled before new practices stabilize [21, 25, 37].

The temporal sequence in the present study is difficult to reconcile with the prediction, common to social-cognitive models of behavior change, that attitude change precedes and drives behavioral change (cf. [17]). The reduction in coercive interventions preceded the measured improvement in attitudes: the sharp decline followed the strategy announcement in 2022, whereas attitude change was assessed in relation to the training in 2023. This pattern suggests that organizational and symbolic change, leadership commitment, shared framing, and increased monitoring, can initiate behavioral shifts before individual attitudes have formally changed. Attitudinal improvement may then serve a consolidating rather than an initiating function, helping to sustain behavioral change by providing cognitive and emotional coherence to new practices. This is consistent with implementation science perspectives that distinguish between early adoption and later internalization [21, 26], and with Vandamme et al. [18], who similarly found that explicit endorsement of change does not guarantee durable behavioral shifts when institutional structures remain unchanged.

Moral psychology further illuminates these dynamics. Judgements about responsibility and deservingness influence whether self-harm is understood as a symptom or as a choice [5, 38]. When self-harm is seen as voluntary or manipulative, irritation and blame can surface, leading to distance or control [2, 16, 18]. By (re-)framing self-harm as a form of emotional regulation rather than as an expression of intent, the regional strategy may have supported cognitive reappraisal, shifted moral judgement, and opened space for empathy [1, 9, 39]. Qualitative work by Thomas and Haslam [40], shows how clinicians describe their work with self-harm as an ongoing balancing act between empathy, responsibility, and self-protection, which resonates with the patterns observed here.

### Structural and organizational mechanisms

The absence of a further decline in coercion after training does not imply that elimination of coercion is a realistic or necessary benchmark. Rather, the stabilization at a substantially reduced level may itself represent a meaningful and sustainable outcome. That coercion did not continue to fall suggests, however, that organizational and emotional structures continued to shape everyday practice beyond what training alone could modify. As Menzies [36] argued, institutions often maintain practices that protect staff from anxiety, even when these practices conflict with stated values. High workloads, staff turnover, and resource constraints can keep coercive routines in place, functioning as a psychological safety net when staff feel overwhelmed [4]. Institutional accountability frameworks tend to privilege what can be counted, rendering relational work comparatively invisible [41, 42].

Recent qualitative studies report similar tensions, pointing to systemic pressures, professional hierarchies, and institutional inertia as barriers to reducing coercion, attributing their persistence partly to the psychological containment coercion provides at the organizational level [43]. In a Danish context, this can be read alongside the notion of a “lean relational space”, where efficiency demands and documentation requirements limit time and legitimacy for relational care [44].

Implementation research underlines that sustainable change depends on leadership, supervision, and consistent feedback loops [15, 25]. Without these supports, emotional labor becomes a solitary task, which increases the risk of burnout and a reversion to control-based norms [4, 18, 35, 45, 46]. Organizational learning frameworks suggest that sustainability arises when data are linked to reflection and when evidence legitimizes new forms of work [15, 25]. Sustaining these reductions likely depends on continued spaces for reflection, embedding relational safety into policies and supervision, and recognizing relational work as legitimate professional activity [47].

### Strengths, limitations, and future directions

This study contributes to a growing body of work that views change in mental health care as both behavioral and organizational. Strengths include the multi-year, system-level dataset, the use of pre-specified intervention points, and the integration of attitudinal and behavioral outcomes. Compared with simple pre–post designs, the interrupted time series approach strengthens causal interpretation by modelling pre-existing trends and temporal alignment with the intervention [23]. The concentration analyses further support the robustness of the observed reductions by showing that results were not driven by a small group of high-frequency patients.

Several limitations must be noted. First, the absence of randomization and of a contemporaneous control series (e.g., other Danish regions or national comparator trends) leaves the analyses vulnerable to unmeasured influences and concurrent initiatives within and beyond the system and limits causal inference [23, 25]. Relatedly, we did not benchmark observed changes against regional or national time trends in coercion; therefore, we cannot determine the extent to which the observed step changes reflect strategy-specific effects versus broader secular developments in coercion reduction.

Second, incomplete denominators, such as patient-days, limited rate calculations and compelled a focus on counts. Changes in bed occupancy, admission patterns, or case-mix over time could therefore have contributed to changes in counts independent of changes in clinical practice.

Third, although the intervention milestones were pre-specified and the ITS design reduces bias relative to simple pre–post comparisons, regression to the mean remains a potential concern if baseline levels of coercion in the early period were unusually high. However, the long pre-intervention observation window and explicit modelling of pre-intervention trends mitigate (but do not eliminate) this threat to inference [23].

Fourth, attrition in SHAS-DR responses likely biased attitudinal findings in a positive direction, as less engaged or less responsive staff were probably more likely to drop out, inflating observed mean improvements. The cross-sectional means in Table 2 should therefore be interpreted as upper-bound approximations, reflecting both within-person change and differential dropout of participants with initially higher antipathy scores. The mixed-effects model in Table 4 provides less biased estimates and should be given most weight; convergence with the paired estimates in Table 3—both showing approximately 3 points improvement—suggests a real effect, though likely overestimated in the descriptive means.

Fifth a related limitation concerns the unit of classification. Patients were classified as self-harming (SHP) or non-self-harming (NSHP) at the patient level rather than at the episode level, that is, based on whether the patient was known to engage in self-harm, not on whether self-harm was the precipitating reason for each specific coercive intervention. This means that coercive episodes involving SHP patients may have been triggered by aggression directed at others rather than by self-harm behavior. As a result, the proportion of coercive episodes attributable to self-harm contexts may be overestimated in the SHP group. It should be noted, however, that the classification procedure included cross-referencing SEI data with coercion protocols and direct staff consultation, which provided some episode-level information about precipitating circumstances. The lack of systematic coding highlights the need for improved documentation to support surveillance and research. This limitation affects the attribution of cause within the SHP group (self-harm vs. aggression as precipitating factor) but does not affect the overall observed reduction in coercive interventions across the study period.

Finally, staff turnover, variation in local implementation fidelity, and the absence of direct observational measures of strategy enactment are additional limitations that could not be fully addressed within the current design.

Despite these limitations, the dual design offers complementary perspectives on psychological and organizational mechanisms of change and aligns with current recommendations for studying complex interventions in routine systems [15, 17, 21, 25, 35, 39].

Future research should examine whether training that explicitly targets emotional labor and moral appraisal can influence long-term behavioral outcomes and whether structural interventions can modify institutional defenses over time [15, 25, 35, 36]. Comparative designs with staggered implementation or matched control sites would strengthen causal inference [23]. Mixed-level analyses linking staff attitudes to coercion data and qualitative inquiry into how cultural change becomes embodied in everyday practice would deepen theoretical understanding [16–18, 39].

### Implications for practice and policy

The findings have several implications for practice and policy. First, training on self-harm should address emotional regulation and moral appraisal explicitly, not only knowledge and procedures [2, 3, 16, 18, 35, 39, 48]. Recognizing emotional labor as part of professional skill may reduce moralization and help protect staff from burnout [4, 35, 36, 45].

Second, structural support is essential. Supervision, peer reflection, and stable staffing are needed to turn individual learning into collective practice and to prevent responsibility for emotional regulation from resting solely with the individual clinician [4, 15, 25, 36].

Third, substitution effects must be monitored. The marked fall in mechanical restraint, combined with more modest change in rapid tranquillization, underscores the need to monitor all forms of coercion to ensure that one form of containment is not simply replaced by another [2, 3, 23]. The sharper reduction in mechanical restraint may reflect modality-specific drivers, such as differences in visibility, normative acceptability, and local implementation focus, rather than differential change in underlying clinical risk. From a policy and ethics perspective, this pattern highlights the importance of making “least restrictive” principles explicit across coercion types and of interpreting changes in coercion profiles in light of patient experience, not only counts [3, 16]. Future evaluations should benchmark local trajectories against national coercion statistics to clarify whether differential reductions represent broader secular trends or strategy-specific change.

Fourth, outcome frameworks should incorporate indicators of unit climate and relational engagement, so that relational care is visible alongside procedural activity, for instance through instruments such as the Ward Atmosphere Scale [49]. The later increase in non-self-harm-related coercion further suggests that approaches developed for self-harm may have wider relevance, and broader restraint-reduction strategies could benefit from integrating similar relational and emotional components.

## Conclusions

The regional self-harm strategy was associated with an immediate reduction in coercive interventions at the time of the strategy announcement, while staff attitudes toward self-harm improved following training and remained more positive eight months later. These effects stabilized rather than continuing to grow. The findings suggest that lasting change requires more than new knowledge: it depends on emotional regulation, moral reframing, and organizational containment. Emotional labor and social-defense theory offer one way to understand why change in mental health care is rarely linear. To become ordinary, new practices must be adopted, repeated, and supported, not only at the level of the individual clinician but within the structures that define everyday care.

## Electronic supplementary material

Below is the link to the electronic supplementary material.

Supplementary Material 1

## Data Availability

The datasets generated and/or analyzed during the current study are not publicly available due to confidentiality agreements with participants but are available from the corresponding author on reasonable request.

## Abbreviations

BPD: Borderline personality disorder
DBT: Dialectical Behavior Therapy
ITS: Interrupted time-series
NSSI: Non-suicidal self-injury
NSHP: Non-self-harming patients
SHP: Self-harming patients
SHAS: Self-Harm Antipathy Scale
