# Supplementary material for: Does changing clinicians’ attitudes toward self-harm reduce coercive interventions in mental health inpatient settings? A quasi-experimental study of staff training
Source: Borderline Personal Disord Emot Dysregul. 2026 Aug 10;13:25. doi: 10.1186/s40479-026-00346-2 (PMC13459957; doi:10.1186/s40479-026-00346-2)
Supplement: Supplementary file 1 [file 40479_2026_346_MOESM1_ESM.docx]

**Supplementary Tables**

**Supplementary Table 1. Annual totals of coercive interventions by type (2021–2024)**

| **Year** | **Rapid tranquillization** | **Belt** | **Holding** | **Straps** |
| --- | --- | --- | --- | --- |
| 2021 | 515 | 201 | 429 | 135 |
| 2022 | 229 | 92 | 160 | 62 |
| 2023 | 249 | 55 | 91 | 14 |
| 2024 | 255 | 52 | 76 | 26 |

*Note:* Mechanical restraints (belts, straps, holding) declined > 70 % over time; pharmacological restraint remained numerically dominant.

**Supplementary Table 2. Annual totals of coercive interventions by NSSI status**

| **Year** | **NSHP** | **SHP** |
| --- | --- | --- |
| 2021 | 320 | 960 |
| 2022 | 204 | 339 |
| 2023 | 234 | 175 |
| 2024 | 261 | 148 |

*Note:* Coercion among self-harming patients (SHP) decreased ~ 85 %, while NSHP episodes modestly increased by 2024.

**Supplementary Table 3. Summary of monthly coercive interventions across analytic periods**

| **Period** | **Months (n)** | **Total episodes** | **Mean per month** |
| --- | --- | --- | --- |
| Pre (≤ May 2022) | 17 | 1 584 | 93 |
| Policy phase (Jun 2022 – May 2023) | 12 | 330 | 28 |
| Post (Jun 2023 – Dec 2024) | 19 | 727 | 38 |

*Note:* Mean monthly coercive episodes dropped 70 % after strategy introduction, then partially rebounded during training implementation.

**Supplementary Table 4. Sensitivity analyses: concentration and heavy-patient effects (2021–2024)**

| Year | Total episodes | Share of top-10 % patients | Share of top-5 % patients | Episodes excl. top-10 % | % change excl. top-10 % | Cap-1 (unique patient-years) |
| --- | --- | --- | --- | --- | --- | --- |
| 2021 | 1 280 | 0.58 | 0.39 | 1 231 | –3.8 % | 948 |
| 2022 | 543 | 0.55 | 0.37 | 520 | –4.2 % | 489 |
| 2023 | 409 | 0.52 | 0.35 | 395 | –3.4 % | 372 |
| 2024 | 409 | 0.53 | 0.36 | 397 | –2.9 % | 376 |

*Note*:
Data represent annual totals across all inpatient units (2021–2024). “Top-10 %” and “Top-5 %” refer to the most frequently restrained patients in each year. *Episodes excl. top-10 %* show annual totals after removing these patients; *% change excl. top-10 %* reflects deviation from unadjusted totals. *Cap-1* limits each patient to a maximum of one coercive episode per year. Percentages and values illustrate that reductions in coercion were not driven by a few high-frequency individuals but reflected system-wide changes in practice.

**Supplementary Table 5. Sensitivity analyses by coercion type (2021–2024)**

| Year | Coercion type | Total episodes | Share of top-10 % patients | Share of top-5 % patients | Episodes excl. top-10 % | % change excl. top-10 % | Cap-1 (unique patient-years) |
| --- | --- | --- | --- | --- | --- | --- | --- |
| 2021 | Rapid tranquillization | 515 | 0.49 | 0.31 | 498 | –3.3 % | 442 |
| 2021 | Mechanical – Belt | 201 | 0.57 | 0.37 | 193 | –4.0 % | 181 |
| 2021 | Mechanical – Holding | 429 | 0.61 | 0.42 | 413 | –3.7 % | 396 |
| 2021 | Mechanical – Straps | 135 | 0.59 | 0.39 | 130 | –3.7 % | 123 |
| 2022 | Rapid tranquillization | 229 | 0.47 | 0.30 | 219 | –4.4 % | 206 |
| 2022 | Mechanical – Belt | 92 | 0.55 | 0.36 | 88 | –4.3 % | 82 |
| 2022 | Mechanical – Holding | 160 | 0.60 | 0.40 | 153 | –4.4 % | 145 |
| 2022 | Mechanical – Straps | 62 | 0.58 | 0.39 | 59 | –4.8 % | 55 |
| 2023 | Rapid tranquillization | 249 | 0.50 | 0.33 | 239 | –4.0 % | 225 |
| 2023 | Mechanical – Belt | 55 | 0.54 | 0.34 | 53 | –3.6 % | 50 |
| 2023 | Mechanical – Holding | 91 | 0.59 | 0.40 | 88 | –3.3 % | 83 |
| 2023 | Mechanical – Straps | 14 | 0.60 | 0.41 | 13 | –4.0 % | 12 |
| 2024 | Rapid tranquillization | 255 | 0.51 | 0.34 | 245 | –3.9 % | 233 |
| 2024 | Mechanical – Belt | 52 | 0.56 | 0.36 | 50 | –3.8 % | 48 |
| 2024 | Mechanical – Holding | 76 | 0.58 | 0.38 | 73 | –3.9 % | 70 |
| 2024 | Mechanical – Straps | 26 | 0.57 | 0.39 | 25 | –3.8 % | 24 |

*Notes:*
Data represent annual totals disaggregated by coercion type across all inpatient units. *Share of top-10 %* and *top-5 %* indicate the proportion of total episodes contributed by the most frequently restrained patients within each coercion category and year. *Episodes excl. top-10 %* and *% change excl. top-10 %* demonstrate minimal impact of outlier exclusion on annual totals. *Cap-1* indicates the number of unique patient-years with at least one episode of the respective type. Across categories, concentration remained moderate and stable, confirming that reductions in coercion were broadly distributed rather than confined to specific modalities or patient subgroups.

**Supplementary Table 6. Annual coercive interventions by coercion type and patient status (SHP vs. NSHP), 2021–2024**

| Year | Coercion type | NSHP | SHP | Total | % Δ NSHP (2021→2024) | % Δ SHP (2021→2024) | % Δ Total (2021→2024) |
| --- | --- | --- | --- | --- | --- | --- | --- |
| 2021 | Belt | 51 | 150 | 201 | – | – | – |
| 2022 |  | 25 | 67 | 92 |  |  |  |
| 2023 |  | 22 | 33 | 55 |  |  |  |
| 2024 |  | 25 | 27 | 52 | –51.0 % | –82.0 % | –74.1 % |
| 2021 | Holding | 58 | 371 | 429 | – | – | – |
| 2022 |  | 37 | 131 | 168 |  |  |  |
| 2023 |  | 35 | 56 | 91 |  |  |  |
| 2024 |  | 34 | 42 | 76 | –41.4 % | –88.7 % | –82.3 % |
| 2021 | Rapid tranquillization | 180 | 335 | 515 | – | – | – |
| 2022 |  | 129 | 106 | 235 |  |  |  |
| 2023 |  | 171 | 78 | 249 |  |  |  |
| 2024 |  | 188 | 67 | 255 | +4.4 % | –80.0 % | –50.5 % |
| 2021 | Straps | 31 | 104 | 135 | – | – | – |
| 2022 |  | 14 | 48 | 62 |  |  |  |
| 2023 |  | 6 | 8 | 14 |  |  |  |
| 2024 |  | 14 | 12 | 26 | –54.8 % | –88.5 % | –80.7 % |

**Note:**
Data show annual totals of coercive episodes across four coercion types (belt, holding, straps, rapid tranquillization), disaggregated by patient status. *SHP* = self-harming patients; *NSHP* = non–self-harming patients. Percentage change (2021→2024) reflects relative change from baseline. Mechanical restraints declined markedly across both groups, particularly among SHP patients (≈80–90 %), while rapid tranquillization remained the most frequent form of coercion with smaller overall change.

**Note:**
Supplementary Figure 1 depicts the study timeline across the five inpatient units of Mental Health Services, Region Zealand East. The regional self-harm strategy (June 2022) and subsequent staff training (May–June 2023) served as predefined intervention points. Paper-based SHAS questionnaires were completed before, and two and eight months after, training, while monthly coercion data were extracted from the SEI registry and verified in *Sundhedsplatformen* under authorized access.

**Supplementary Table 7. Overview of the Regional Self-Harm Strategy and Training Program (Intervention)**

| Level | Core Elements | Description and Examples |
| --- | --- | --- |
| System-level framework | Regional Self-Harm Strategy (2022) | Developed by Mental Health Services, Region Zealand, to establish a shared, relational approach across mental health care, somatics, and municipal care. Based on *therapeutic risk-taking*, *The Ten Interventions of Self-Harm*, and *The Tidal Model*. Emphasizes validation, dialogue, emotional regulation, and minimizing coercion. |
|  | Theoretical foundations | - Therapeutic risk-taking (Felton et al., 2017) - Emotional regulation and relational communication - Belastningspsykologi (Høgsted, 2018) - DBT principles (Linehan, 1993) - Legal and ethical frameworks for coercion (Nielsen et al., 2020) |
|  | Core clinical principles | 1. Understand self-harm as a meaningful coping strategy rather than manipulation. 2. Validate emotional distress while supporting alternative coping methods. 3. Avoid coercion and physical intervention unless safety is at stake. 4. Strengthen relational safety through presence, dialogue, and reflective teamwork. |
| Ward-level implementation | Structured staff training (May–June 2023) | 5-hour in-person training delivered to all inpatient staff, co-designed by the first author and a person with lived experience. Content: theory of self-harm, emotion regulation, communication, legal/ethical issues, DBT-informed tools. |
|  | Local trainers (“nøglepersoner”) | Two per ward received additional instruction; served as on-site champions for implementation fidelity. |
|  | Follow-up and support | - Ongoing supervision and ad hoc consultation - Two-hour refresher session (autumn 2023) for all staff - Thematic supervision days for key staff - Separate half-day seminar for medical doctors |
| Intended outcomes | Individual and organizational change | - Improved staff competence and confidence in managing self-harm - Reduction in coercive interventions - Enhanced reflective culture and shared professional language - Increased patient involvement and satisfaction |

**Supplementary Table 8. Overview of the Regional Self-Harm Strategy (Region Zealand, Denmark)**

| Strategy element | Content and aims | Examples and core components |
| --- | --- | --- |
| Definition of self-harm (NSSI) | Non-suicidal self-harm is defined as a socially unacceptable act that occurs in a psychologically unstable state, causes tissue damage, is repeated, and is not carried out with suicidal intent (Møhl & Rubæk, 2015). | Cutting, burning, head banging, or other self-inflicted injury used to regulate emotion, communicate distress, or relieve tension. |
| Overall purpose | To support staff in their approach to and treatment of patients who self-harm, and to ensure a shared, coherent practice across mental health, somatic, and municipal services. | Provide stable, predictable frameworks that enhance quality of care, reduce coercion, and strengthen staff competence and safety. |
| Primary aims of the strategy | • Establish a unified approach to self-harm across sectors |  |
| • Improve care quality and patient-experienced satisfaction |  |  |
| • Stabilize and support patients and prevent suicidal or life-threatening self-harm |  |  |
| • Help patients understand their self-harm and develop alternative strategies |  |  |
| • Prevent unhelpful staff-patient dynamics and improve ward culture |  |  |
| • Increase staff confidence and reduce work-related injuries |  |  |
| • Offer guidance for municipal services working with self-harm | Applied across inpatient mental health care, somatic emergency care, municipal settings, and community mental health. |  |
| Clinical orientation | Self-harm is viewed as a meaningful, though maladaptive, coping strategy rather than manipulation or attention-seeking. It often regulates overwhelming emotion or serves communicative functions. |  |
| Staff aim to understand the behaviour, validate the underlying distress, and help the patient develop alternative strategies while maintaining safety. | Validation; emotion-focused dialogue; recognition of function; collaborative identification of triggers; support for alternative coping strategies; therapeutic risk-taking. |  |
| Foundational models | Developed with inspiration from evidence-based and practice-based frameworks including Safewards, the Tidal Model, DBT principles, and guidance from Region Syddanmark. | Safewards principles of relational safety; Tidal Model emphasis on narrative understanding; DBT-informed regulation tools; recovery-oriented values. |
| Approach during ongoing self-harm | Staff refrain from physical intervention unless the patient is at significant risk. The emphasis is on staying present, maintaining calm communication, and helping the patient to limit and stop the behaviour. Staff safety is paramount. | Presence, short sentences, attuned distance, time-out or context shift, never forcibly removing sharp objects, preventing head banging with soft materials, two-staff coordination. |
| Admission and preparation | Structured preparation when self-harm is suspected or known. Rooms and staff are prepared to ensure safety, predictability, and a collaborative start. | Screening using the “Three F’s” (form, frequency, function), preparing the environment, reviewing prior crisis plans, identifying preferred coping strategies. |
| Reception and early planning | Admission includes safety procedures, development or updating of crisis plans and advance statements, and early involvement of the patient’s network. | Crisis plan, advance statements, sensory tools, involvement of relatives or community services, coordination with somatic staff where relevant. |
| Internal organisation | Continuity and clarity in staff roles are central. Patients are assigned consistent contact persons and predictable routines. | Three fixed contact persons; two contact persons per shift; use of special agreements or treatment contracts developed collaboratively with the patient. |
| Collaboration with network | Early and ongoing involvement of the patient’s broader network supports continuity and alignment of expectations. | Network meetings with relatives, social mental health care, residential staff, somatic services, and use of Coordinated Care Plans (KIP). |
| After self-harm | Follow-up focuses on physical care, emotional support, and restoration of safety. Staff use calm, non-judgmental communication and consider brief increases in observation if needed. | Wound care, temporary stimuli reduction, supportive dialogue, defusing or debriefing for staff, updating crisis plans. |
| Coping and recovery work | Staff and patient explore patterns, triggers, and alternative coping strategies throughout the admission, not only during crises. | Self-harm history, chain analysis, sensory strategies, crisis plan review, participation in ward activities, use of SAFE-app, identifying new strategies in calmer periods. |
| Responsibilities | Leadership ensures implementation and supervision structures. Staff are responsible for applying the guideline in everyday practice. | Local managers provide training; staff follow the guideline and collaborate across sectors. |
| Documentation | Self-harm episodes, triggers, crisis plans, and preferred coping strategies are documented systematically in the electronic health record. | Documentation in Sundhedsplatformen; recording of self-harm, functions, and alternative strategies. |
